# Inactivation of the Autolysis-Related Genes *lrgB* and *yycI* in *Staphylococcus aureus* Increases Cell Lysis-Dependent eDNA Release and Enhances Biofilm Development *In Vitro* and *In Vivo*

**DOI:** 10.1371/journal.pone.0138924

**Published:** 2015-09-25

**Authors:** Cristiana Ossaille Beltrame, Marina Farrel Côrtes, Raquel Regina Bonelli, Ana Beatriz de Almeida Côrrea, Ana Maria Nunes Botelho, Marco Antônio Américo, Sérgio Eduardo Longo Fracalanzza, Agnes Marie Sá Figueiredo

**Affiliations:** Universidade Federal do Rio de Janeiro, Instituto de Microbiologia Paulo de Góes, Departamento de Microbiologia Médica, Rio de Janeiro, RJ, Brazil; Institut Pasteur, FRANCE

## Abstract

*Staphylococcus aureus ica*-independent biofilms are multifactorial in nature, and various bacterial proteins have been associated with biofilm development, including fibronectin-binding proteins A and B, protein A, surface protein SasG, proteases, and some autolysins. The role of extracellular DNA (eDNA) has also been demonstrated in some *S*. *aureus* biofilms. Here, we constructed a Tn*551* library, and the screening identified two genes that affected biofilm formation, *lrgB* and *yycI*. The repressive effect of both genes on the development of biofilm was also confirmed in knockout strains constructed by allelic recombination. In contrast, the superexpression of either *lrgB* or *yycI* by a cadmium-inducible promoter led to a decrease in biofilm accumulation. Indeed, a significant increase in the cell-lysis dependent eDNA release was detected when *lrgB* or *yycI* were inactivated, explaining the enhanced biofilm formed by these mutants. In fact, *lrgB* and *yycI* genes belong to distinct operons that repress bacterial autolysis through very different mechanisms. LrgB is associated with the synthesis of phage holin/anti-holin analogues, while YycI participates in the activation/repression of the two-component system YycGF (WalKR). Our *in vivo* data suggest that autolysins activation lead to increased bacterial virulence in the foreign body animal model since a higher number of attached cells was recovered from the implanted catheters inoculated with *lrgB* or *yycI* knockout mutants.

## Introduction

Methicillin-resistant *Staphylococcus aureus* (MRSA) has entered the spotlight as a globally spread community- and health care-associated pathogen. During its evolutionary path toward becoming a successful human pathogen, in addition to the ability to develop biofilms, *S*. *aureus* has acquired an assortment of virulence mechanisms and refined strategies to evade the host immune system that are associated with producing an overabundance of surface proteins and exoproteins [1–4]. Biofilm development has been recognized as an important virulence attribute for the pathogenesis of intravenous catheter-related bacteremia and infections associated with the use of medical prostheses [1, 2, 5, 6]. Two types of biofilms have been described in *S*. *aureus* thus far: the *ica*-dependent biofilm, associated with polysaccharide intercellular adhesin/poly-N-acetylglucosamine (PIA/PNAG; product of the *ica* operon), and the *ica*-independent biofilm [4, 7]. Recent studies have suggested that *ica*-independent biofilms have a proteinaceous matrix and that they are more frequently found among MRSA isolates [8, 9]. Surface proteins referred to as MSCRAMMs (microbial surface component-recognizing adhesive matrix molecules) appear to be the primary determinant for the initial attachment to both biotic and abiotic surfaces [8]. Some proteins such as protein A (Spa), fibronectin-binding proteins A and B (FnBPA and FnBPB), and *S*. *aureus* surface protein G (SasG), among others, have been associated with *ica*-independent biofilm formation and accumulation [8–11].

Biofilms represent a unique growth environment where several loci can be differentially expressed, as some virulence regulatory genes are, for instance [4, 12]. The *agr* operon, encoding the major *S*. *aureus* quorum-sensing system, has been shown to play a role in the modulation of biofilms [7, 13]. This system downregulates genes involved in host colonization such as those encoding for some MSCRAMMS (including FnBPAB and Spa), and upregulates those encoding for some exoproteins (e.g., α-hemolysin-Hla and phenol soluble modulin α-Psmα) involved in tissue damage. Recently, studies with *agr*-null mutants derived from contemporary MRSA clinical isolates showed that the effect of *agr* on *ica*-independent biofilms varied from negative or neutral to positive [13]. It was reported that the transcriptional regulator SarA and the two-component system (TCS) SaeRS have a positive impact on the development of *ica*-independent biofilms, which seems to be related to the repression of extracellular proteases by SaeRS [12, 14, 15].

In addition to proteins, extracellular DNA (eDNA) provided by the autolysis of subsets of sessile and planktonic cells is an important compound of the biofilm matrix, and it seems to play a role either in the initial surface attachment or the intercellular aggregation during biofilm maturation [16, 17]. More recently, proteins exhibiting characteristics similar to those of holin/anti-holin phage proteins have been implicated in the induction/repression of the bacterial autolytic process [18–20]. A knockout derived from the *S*. *aureus* UAMS-1 strain for inactivating the *cidA* gene, which codes for a protein analogous to phage holin, resulted in the reduced activation of murein hydrolases, the release of eDNA, and the bacterial ability to accumulate biofilm, while a mutation in the *lrgAB* operon encoding for an anti-holin-like protein had the opposite effect [19–21].

Although some progress has been made in elucidating the key components involved in the modulation of *ica*-independent biofilms, these studies are still in their early stages, and the genetic manipulations have generally been performed with laboratory strains such as the 8325–4 derivatives that contain a series of additional mutations. Consequently, the use of such isolates may compromise the accuracy of the data produced, since they may not correctly reproduce the true effects of inactivating a given locus in the *S*. *aureus* genome [22].

The aim of this study was to identify genes involved in the modulation of *ica*-independent biofilms through screening a isogenic Tn*551* insertion library with a *S*. *aureus* clinical isolate from the ST5 lineage as the wild-type (WT) strain. This work also aims to correlate the results of the in vitro biofilm assay with that of murine foreign body infection model in order to provide a better understanding of the role of the identified genes on the development of medical-device related infections.

## Material and Methods

### Ethics Statement

This project was approved by the Human Research Ethics Committee from Clementino Fraga Filho, Universidade Federal do Rio de Janeiro (Protocol #IMPG 018). Participant consents could not be obtained because the authors have only received the bacterial isolates from the microbiology laboratory after the patients have been discharged from the hospitals. In addition, the authors declare that the samples were anonymized before receiving them, and that they have no access to any data that would put patient privacy and security at risk. The authors also state that the lack of consents was approved by the ethics committee, and that this study was conducted according to the principles expressed in the Declaration of Helsinki. The animal protocol was approved by the Ethics Committee for Animal Care and Use at the Federal University of Rio de Janeiro (Protocol #055/14).

### Bacterial strain and plasmid/genetic constructions

Tables 1 and 2 list the bacterial plasmids, strains, and constructions that were generated from the *S*. *aureus* WT strain HC474. This strain was collected from a patient admitted to a home-care system in the city of Rio de Janeiro, and it accumulates a moderate amount of *ica*-independent biofilm on polystyrene surfaces. The isolate is genetically related to the New York/Japan and pediatric clones (USA100/USA800; sequence type 5) circulating in hospitals worldwide. In addition, for some experiments of gene expression, the MRSA clinical isolates BMB9393 and GV69 were also included. All bacterial strains were stored in 12% glycerol at −80°C.

**Table 1:** Most relevant strains used in this study.

Strain	Description	Designation	Reference
***Staphylococcus aureus***			
HC474	ST5, Eri^S^, moderate biofilm producer	WT	13
M1069	HC474Ω*yycI*::Tn*551*	*yycI*::Tn*551*	This work
M1321	HC474Ω*lrgB*::Tn*551*	*lrgB*::Tn*551*	This work
M1HC474	HC474Ω*lrgB*::pLGEM	*lrgB*::pLGEM	This work
M2HC474	HC474Ω*yycI*::pYGEM	*yycI*::pYGEM	This work
C1HC474	HC474ΩlrgB::pLGEM; pLCN42:P_cad_ *-lrgB*	P_cad_ *-lrgB*	This work
C2HC474	HC474Ω*yycI*::pYGEM; pLCN42:P_cad_ *-yycI*	P_cad_ *-yycI*	This work
RN9598	RN4220 transformed with pCN42		30
BMB9393	ST239-SCC*mec*III, strong biofilm producer		13
GV69	ST239-SCC*mec*III, moderate biofilm producer		13
***Escherichia coli***			
DC10B	*E*. *coli* DH10BΔ*dcm*		29

**Table 2:** Plasmids used in this study.

Plasmid	Description	Resistance	Reference
pRN3208	Thermosensitive for replication, and carrying Tn*551*	Ery	23
pSK265	*S*. *aureus* plasmid used as template for *cat* amplification	Cm	28
pGEM *T-easy* vector	Used for cloning assays	Amp	Promega
pLGEM	pGEM: _(5′)_ *lrgB-cat*-_(3′)_ *lrgB*	Amp (*E*. *coli*) Cm (*S*. *aureus*)	This work
pYGEM	pGEM: _(5′)_ *yycI-cat*-_(3′)_ *yycI*	Amp (*E*. *coli*) Cm (*S*. *aureus*)	This work
pCN42	*E*.*coli*-*S*. *aureus* shuttle vector with a cadmium inducible promoter	Ery	30
pLCN42	pCN42:P_cad_-*lrgB*	Ery	This work
pYCN42	pCN42:P_cad_-*yycI*	Ery	This work

Cm, chloramphenicol; Amp, ampicillin; Ery, erythromycin.

### Screening the Tn*551* mutants with altered biofilm phenotypes

The plasmid pRN3208 was the thermosensitive vehicle used to deliver Tn*551* into the HC474 strain via transduction with phage 80α [23]. The selection of the Tn*551* insertional mutants was performed on plates with 100 μg/ml erythromycin (Sigma; St. Louis, MO, USA) and confirmed by polymerase chain reaction (PCR) using the primers Tn*551*-F and Tn*551*-R (Table A in S1 File). Those insertional mutants that exhibited differential *ica*-independent biofilm phenotypes were then identified with a microtiter-plate screening method as described by Ferreira et al. [24].

### Identifying the genes inactivated by the Tn*551* insertion

The chromosomal site disrupted by the Tn*551* insertion was identified by sequencing the product of a semi-random PCR performed as previously described [25] with the arbitrary M13 primer [26] and a specific primer that anneals in the 5′ (Tn*551*JOUT) or 3′ (Tn*551*ROUT) terminal end of Tn*551* [27] (Table A in S1 File). The PCR products were purified with the QIAquick PCR Purification kit (Qiagen; Hilden, Germany), and their DNA sequences were obtained using a MegaBACE 1000 automatic sequencer (Amersham Biosciences/GE Healthcare; Freiburg, Germany) following the manufacturer recommendations. The sequences were multiply aligned using blastx (www.uniprot.org).

### Gene inactivation by allelic recombination

The transposon-tagged genes identified as *lrgB* and *yycI* were also disrupted by insertional allelic recombination as described previously [9]. Briefly, inserts containing the *cat* gene flanked by homologous 5′ and 3′ fragments of the gene were constructed by PCR amplification with the forward and reverse primers for “*lrgB* knockout” or “*yycI* knockout” to inactivate *lrgB or yycI*, respectively (Table A in S1 File). The *cat* gene was amplified with the primers listed in Table A in S1 File using pSK265 [28] as template (Table 2). The PCR-based constructions were cloned into the pGEM (Promega) *Escherichia coli* vector and the resultant suicide vectors (pLGEM and pYGEM; Table 2) were electroporated into *E*. *coli* DC10B (kindly given by Dr. Timothy Foster, Trinity college, Dublin, Ireland) as described [29]. After selection on 10 μg/ml chloramphenicol plates, the recombinant plasmids were recovered using the QIAfilter Plasmid Midi kit (Qiagen) and subcloned into HC474 electrocompetent cells as previously described [29]. The confirmation of the suicide vector insertion sites was performed by PCR (Table A in S1 File) using an internal primer for the *cat* gene (primer *cat*) and an external target gene sequence adjacent to the insert insertion site (primer *lrgB*-ext or *yycI*-ext). The *lrgB* and *yycI* knockouts were designated M1HC474 (Ω*lrgB*::pLGEM) and M2HC474 (Ω*yycI*::pYGEM), respectively.

### Growth curves

To investigate whether or not the knockout mutants showed significant growth impairments, the growth curves of the WT strain and isogenic knockouts (M1HC474 or M2HC474) were monitored and the doubling times determined using the software Doubling Time Calculator (www.doubling-time.com/compute.php). The results are the mean of three independent experiments. Additionally, since cadmium was used for promoter induction in the complementation experiments, the bacterial growth was also monitored using broth with and without supplementation with 10 μM cadmium.

### Complementation experiments

To restore gene expression in the M1HC474 and M2HC474 mutants, an 830 bp or 850 bp fragment containing the coding region of the *lrgB* or *yycI* genes, respectively, was amplified from HC474 DNA template using specific primers (Table A in S1 File) and Platinum High Fidelity DNA polymerase (Invitrogen; Carlsbad, CA, USA). The DNA fragment was cloned downstream of the cadmium-inducible promoter (P_cad_) in the pCN42 *S*. *aureus* vector [30], a gift from Richard Novick, NYU, USA, resulting in pC1HC474 (P_cad_-*lrgB*) or pC2HC474 (P_cad_-*yycI*). The respective complementation vectors were electroporated into DC10B competent cells [29] and then subcloned by electroporation into M1HC474 or M2HC474, resulting in the trans-complementing constructions C1HC474 and C2HC474, respectively. In addition, for controlling the complementation system and excluding a possible effect of cadmium in biofilm development, an empty pCN42 vector was cloned into M1HC474 and M2HC474. The transcription of each complemented gene in its respective trans-complementing construction was confirmed by real-time quantitative reverse transcription-PCR (real-time qRT-PCR) using the Power SYBR Green RNA-to-CT™ 1-Step kit (Applied Biosystems; Foster City, CA, USA). The results were analyzed by the ΔΔCt comparative method with the Step One software v2.2 as recommended by the manufacturer (Applied Biosystems). The RNA was prepared using the RNeasy Kit (Qiagen; Germantown, MD, USA); the specific primers are listed in Table A in S1 File. To confirm that the knockouts and trans-complementing mutants were closely related to HC474, the clonal complex for each construction was confirmed using restriction-modification tests [31].

### *In vitro* biofilm assay

The role that the transposon-inactivated genes played in the development of *ica*-independent biofilms was further examined in the knockout and trans-complementing isogenic mutants using a microtiter plate-based assay (Nunclon; Nunc A/S, Roskilde, Denmark) with trypticase soy broth (TSB) (BD; Becton, Dickinson and Company, Franklin Lakes, NJ, USA) supplemented with 1% (w/v) glucose (Sigma) as previously described [24]. For testing biofilm development in the complementation experiments, cadmium was added to a final concentration of 10 μM for induction of the P_cad_ promoter [30]. In some experiments, the preformed biofilm was treated with sodium metaperiodate (10 mM/well; Sigma), proteinase K (6 U/well; Invitrogen), or DNase I (56 U/well; Sigma) before washing and subsequent staining with crystal violet. In addition, the differences in biofilm accumulation among the isogenic mutants and WT strain were visualized by confocal laser scanning microscopy (CLSM) as previously described [13]. Because biofilms are under phase-variation, the results are the mean of at least three independent experiments with four replicates.

### Murine foreign body infection model

The animal experiments and protocols were performed as recently described by Ferreira et al. [24]. Animals receive food and water *ad libitum*. Each cage housed a maximum of 2 mice. Briefly, to analyze the impact of *lrgB* and *yycI* on biofilm development in vivo, the lumen of a 1-cm segment of a subcutaneous polyurethane catheter segments (C-UDLM-953J model; Cook Medical, Bloominaton, USA) inoculated with mid-exponential growth phase culture (10^6^ CFU/10 μl) of M1HC474, M2HC474 or isogenic wild-type HC474 strain was surgically implanted in the back of adult Balb/c male mice (weighing approximately 30 g and aged 6 to 8 weeks). Mice were anesthetized with ketamine (40 mg/kg of body weight) and thiopental (80 mg/kg of body weight). Their flanks were shaved, and the preoperative skin cleansing with tincture of iodine before the surgery. The incision was sutured and disinfected with iodine. The animals were euthanized after three days post-infection, and the catheter segments were surgically removed to assess the biofilm by counting catheter-adherent bacteria by CFU determination. A total of 3 catheters were implanted for each mutant and the isogenic wild-type HC474 in three different animals, and the counting of adherent cells was performed in triplicates.

### Autolysis assay

Triton X-100 induced autolysis assays were performed essentially as described previously with the following modifications [16, 32]. Cultures of *S*. *aureus* were inoculated into 20 ml of fresh brain hearth infusion (BHI) (BD) broth and incubated at 37°C under 200 rpm until an approximate OD_600_ = 1.0 was obtained. Cultures were centrifuged for 10 min at 6.800 x g. Pellets were washed in the same volume of cold water and centrifuged again for 10 min. Pellets were then resuspended in 1 ml of cold water and the OD600 was adjusted to one in 5 ml of water containing 0.1% Triton X-100 (Sigma). The suspensions were mixed by vortex for 10 s and the OD600 of the culture at time zero was recorded. Cultures were then incubated at 37°C under 200 rpm for 4 h and the OD600 was measured every 15 min. The results are the average of three independent experiments.

### Quantification of eDNA

eDNA was quantified in biofilm supernatants using Qubit® 2.0 Fluorometer (Invitrogen; Eugene, Oregon, USA), after ethanol precipitation [33]. The results are the means of two independent experiments with duplicates.

### Gene expression analysis

Total RNA was obtained using an RNeasy Kit (Qiagen) from the sessile cells cultured in vitro for the WT strain HC474, the respective *lrgB*/*yycI* knockout mutants, and for the MRSA clinical isolates BMB9393 and GV69. The primers for the real-time qRT-PCR analyses of the *lrgB*, *yycI*, *fnbA/B*, *sarA*, and *agr*-*rnaIII* genes, as well as the 16S RNA gene used as an endogenous control, are listed in Table A in S1 File. Additionally, total RNA was isolated from free-leaving cells of HC474 strain grown until the beginning of log phase (OD_600_ = 0.3) in TSB supplemented with glucose. The real-time qRT-PCR was performed using the Step One™ Real-Time PCR System (Applied Biosystems), and the data were analyzed with the Step One software v2.2 (Applied Biosystems) as described for the complementation experiments. The results are the means of two independent experiments with triplicates.

### Statistical tests

The Student´s *t* test was used to assess differences in biofilm values between mutant and the wild type strains, and in the experiments of biofilm disruption after treatment with DNase I, protease or sodium metaperiodate. To compare the autolysis kinetics, each curve was fit using non-linear regression, and the best-fit value for each time point was tabulated and compared using paired-*t* test. The significance level chosen was 0.05.

## Results

### Screening of the transposon-tagged genes exhibiting different biofilm accumulation phenotypes

A total of 4603 Tn*551* insertional mutants derived from the HC474 strain were screened, two of which (designated M1069 and M1321) showed increased biofilm accumulation in comparison with the WT. The sequencing of the semi-random PCR products using template DNA from these mutants identified two independent loci disrupted by the Tn*551* insertion, *lrgB* for M1069 (Ω*lrgB*::Tn*551*) and *yycI* for M1321 (Ω*yycI*::Tn*551*). The LrgB protein is analogous to phage anti-holin proteins belonging to a family of orthologs that prevent membrane depolarization or the formation of holes by holin-promoting bacterial cell autolysis [34]. The *yycI* gene negatively regulates the TCS YycG/YycF (also known as Walk/WalR), which is conserved in low G+C content in Gram-positive bacteria. Interestingly, this system is also involved in peptidoglycan biosynthesis, regulating cell division, and autolysis [35].

### Increase in biofilm accumulation for the *lrgB* and *yycI* knockout mutants

**Fig 1:**
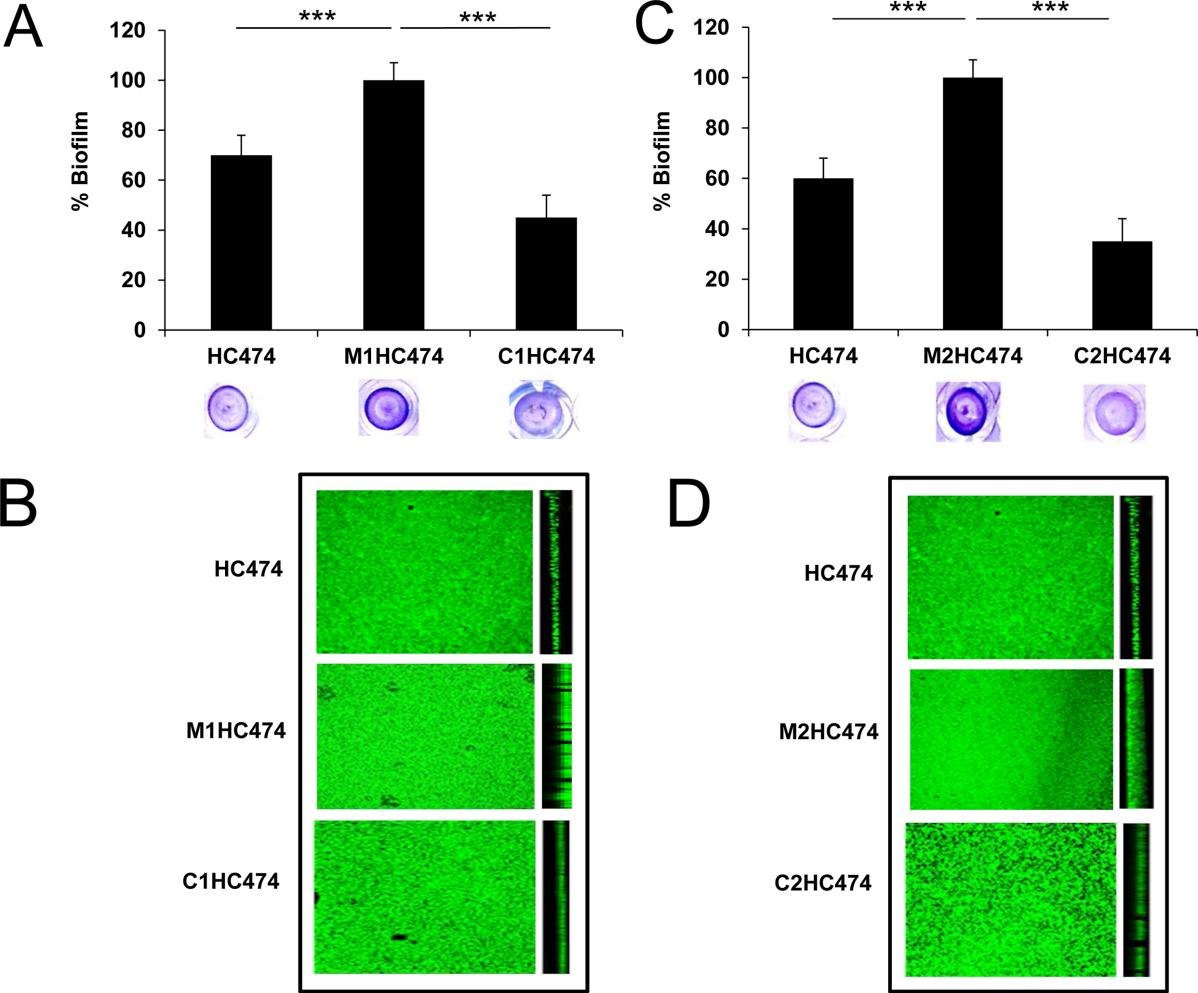
Biofilm development by the wild type (WT) strain HC474, knockout mutants for *lrgB* (M1HC474) and *yycI* (M2HC474) and respective complemented mutants (C1HC474 and C2HC474). (A) Percentage of biofilm accumulation on inert polystyrene surfaces by HC474 (WT), M1HC474 (*lrgB*::pLGEM), and C1HC474 (P_cad_-*lrgB*). (B) Images obtained by confocal laser scanning microscopy (CLSM) of the biofilms accumulated by the WT HC474 (14.10 μm thick), M1HC474 (18.13 μm thick), and C1HC474 (6.04 μm thick). (C) Percentage of biofilm accumulated on inert polystyrene surfaces by HC474 (WT), M2HC474 (*yycI*::pYGEM), and C2HC474 (P_cad_-*yycI*). (D) Images obtained by CLSM of the biofilms accumulated by HC474 (14.10 μm thick), M2HC474 (24.19 μm thick), and C2HC474 (8.06 μm thick). For graphic presentation purposes, biofilm data were transformed into a percentage taken as reference (defined as 100%) the mean value obtained for M1HC474 (A) or M2HC474 (C). ***p < 0.0001.

Relative to the HC474 WT strain, the semi-quantitative examination of the biofilms formed in vitro by the *lrgB* and *yycI* knockouts confirmed extremely significant increases in biofilm accumulation of 30% (*p* < 0.0001) and 40% (*p* < 0.0001) for the M1HC474 (Ω*lrgB*∷pLGEM) and M2HC474 (Ω*yycI*::pYGEM) mutants, respectively (Fig 1). The CLSM images clearly confirmed the increase in biofilm thickness for M1HC474 and M2HC474 in relation to the HC474 WT strain and complemented constructions (Fig 1B and 1D). The increased development of biofilm could not be attributed to possible growth rate differences between the mutants and WT, since no significant differences were detected in the lag phase or doubling time (DT) between the WT and respective mutants [DT_HC474_ = 57.79 ± 3.33 min; DT_M1HC474_ = 60.16 ± 10.76 min; and DT_M2HC474_ = 55.69 ± 4.01].

Confirming the role played by *lrgB* and *yycI* in impairing biofilm accumulation in the HC474 background, the effects of the Ω*lrgB*∷pLGEM and Ω*yycI*∷pYGEM mutations in increasing the biofilm accumulation were successfully reversed, under cadmium induction, in their respective complemented mutants, whose biofilm accumulations were even lower than that observed for the WT strain (Fig 1).

The expression of *lrgB* and *yycI* was also quantified in the respective complementations in the presence of cadmium. The decrease in biofilm accumulation was consistent with the superexpression of *lrgB* or *yycI* in their respective complemented constructions [C1HC474 (Ω*lrgB*::pLGEM; P_cad_ *-lrgB*) and C2HC474 (Ω*yycI*::pYGEM; P_cad_-*yycI*)] in comparison with WT, as confirmed by real-time qRT-PCR using HC474 RNA as the reference (RQ_HC474_ = 1, RQ_C1HC474_ = 61.2 ± 3.55; RQ_C2HC474_ = 55.3 ± 11.1). As expected, C1HC474 and C2HC474 showed very low levels of transcriptional expression (RQ = 0.5 ± 0.56 and RQ = 0.4 ± 0.7, respectively) when cultivated in the absence of cadmium. In parallel, when the promoter was inactive, an extremely significant increase (*p* < 0.0001) in the amount of biofilm developed by both the C1HC474 and C2HC474 strains was detected, further confirming the effect of the mutated genes on *S*. *aureus* biofilm (Data not shown). Essentially, biofilm development in the presence of cadmium by M1H474 or M2H474 cloned with empty pCN42 was not reduced to the levels of C1HC474 or C2HC474, respectively, demonstrating that functional activities of LrgB/YycI, and not an effect of the vector or cadmium, are responsible for the decrease in biofilm mass accumulation detected for the knockout mutants (data not shown).

**Fig 2:**
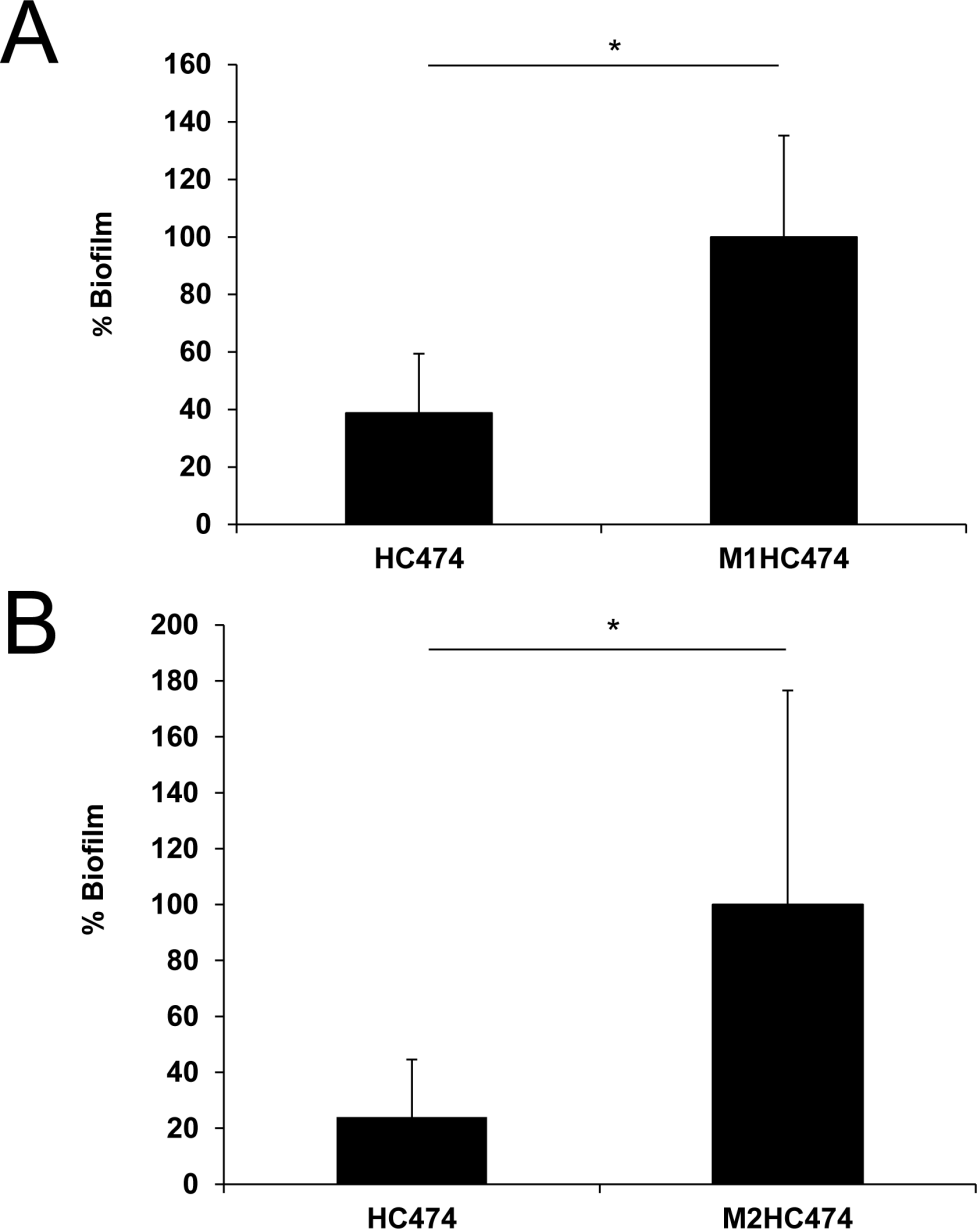
Virulence comparisons between the *S*. *aureus* wild type (WT) strain and the *lrgB* or *yycI* isogenic knockouts in a foreign-body infection model. Difference in the number of bacterial cells recovered from the catheters implanted in mice inoculated with WT (HC474) or (A) M1HC474 (*lrgB*::pLGEM) or (B) M2HC474 (*yycI*::pYGEM). Columns represent the mean number of sessile cells recovered from the catheters implanted in the animals. For graphic presentation purposes, CFU/mL was transformed into a percentage taken as reference (defined as 100%) the average CFU/mL value recovered for (A) M1HC474 or (B) M2HC474. *p < 0.01.

The ability of the M1HC474 and M2HC474 knockouts to accumulate more biofilm was also tested in an infection mouse model. Similarly to what occurred in vitro (Fig 1), both mutants showed a significant superior capacity to accumulate biofilms in the implanted catheters in relation to the wild-type strain (Fig 2). Comparable results were obtained in the vitro experiments, in which more cells were recovered from catheters inoculated with M2HC474 (Fig 2B) in relation to those inoculated with M1HC474 (Fig 2A).

### The effect of proteinase K, DNase I, and sodium metaperiodate on the biofilms formed by HC474 and the isogenic *lrgB* and *yycI* knockouts

**Fig 3:**
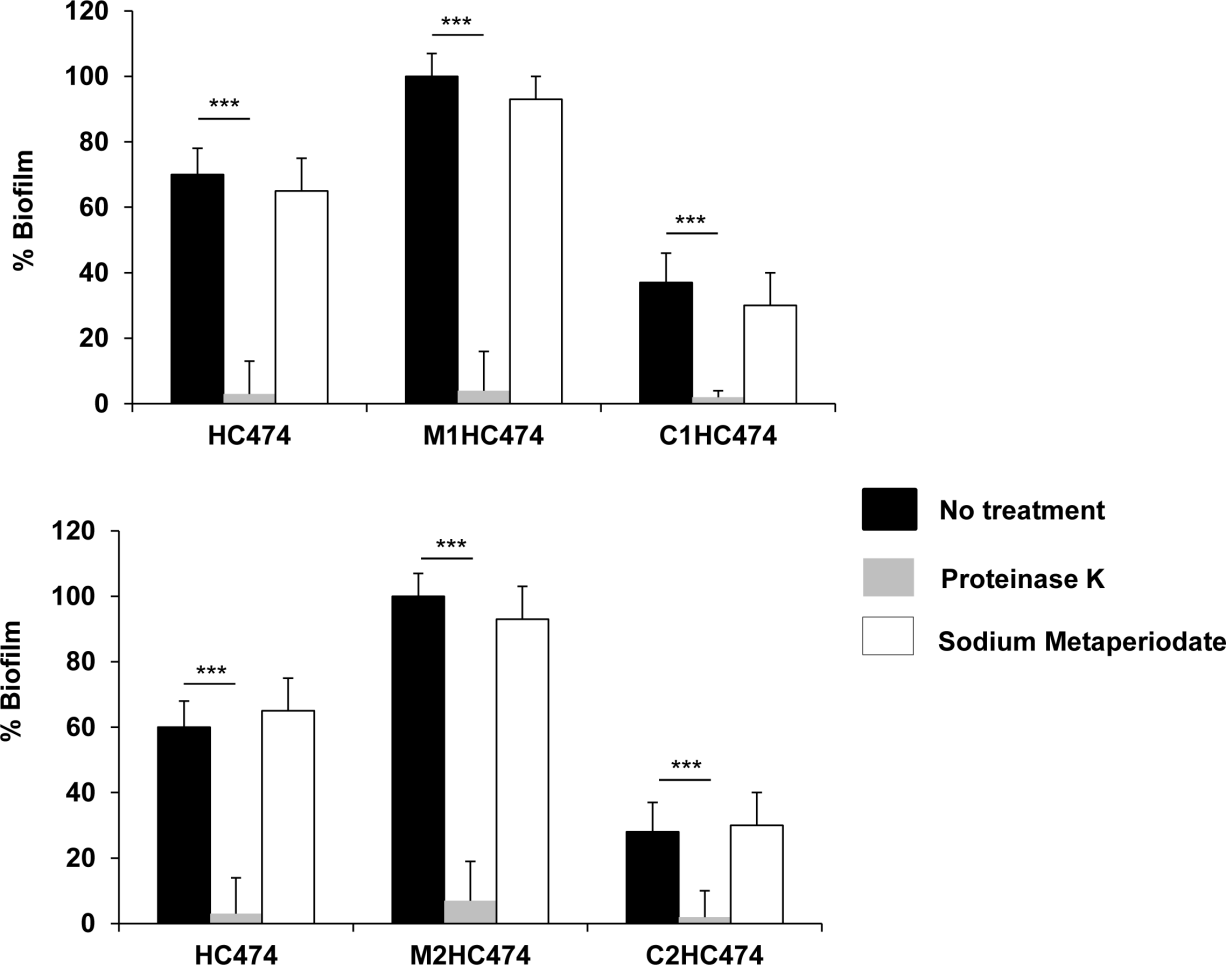
The effects of 10 mM sodium metaperiodate or 6 U of proteinase K on the preformed biofilms developed by the wild type (WT) strain HC474 and the derivative *lrgB* and *yycI* knockout and complemented strains. (A) HC474 (WT), M1HC474 (*lrgB*::pLGEM), and C1HC474 (P_cad_-*lrgB*). (B) HC474 (WT), M2HC474 (*yycI*::pYGEM), and C2HC474 (P_cad_-*yycI*). For graphic presentation purposes, the amount of biofilm was transformed into a percentage taken as reference (defined as 100%) the mean value obtained for M1HC474 (upper panel) or M2HC474 (bottom panel). ***p < 0.0001.

The treatment with sodium metaperiodate did not significantly affect the biofilm accumulated by the WT strain HC474, the *lrgB* and *yycI* knockouts, or the respective trans-complementing mutants, indicating a minor role for the polysaccharide PIA/PNAG in these biofilms. However, proteinase K virtually disrupted the biofilms formed by WT and the strains with the Ω*lrgB*::pLGEM and Ω*yycI*::pYGEM knockout constructions. Similar effects were observed for the respective complemented mutants (Fig 3).

**Fig 4:**
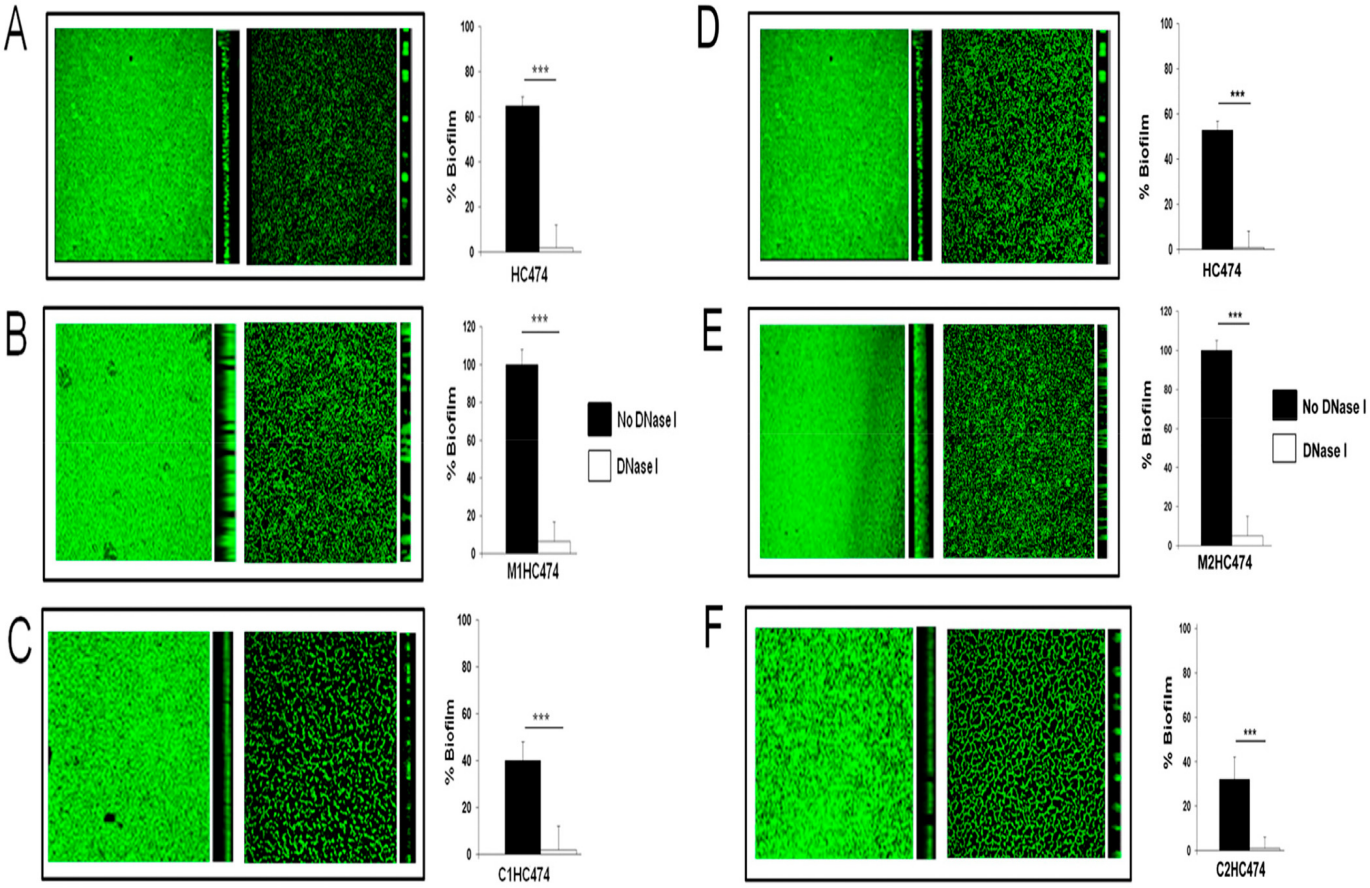
The effects of 56 U of DNase I on preformed biofilms developed by the wild-type (WT) HC474 strain and its derivative *lrgB* and *yycI* knockout and complemented strains. Confocal laser scanning microscopy of biofilms before (left panels) and after (right panels) DNase I treatment using a microtiter-based method. (A) HC474 (WT); (B) M1HC474 (*lrgB*::pLGEM); (C) C1HC474 (P_cad_-*lrgB*); (D) HC474; (E) M2HC474 (*yycI*::pYGEM); and (F) C2HC474 (P_cad_-*yycI*). For graphic presentation purposes, the amount of biofilm was transformed into a percentage taken as reference (defined as 100%) the mean value obtained for M1HC474 or M2HC474. ***p < 0.0001.

Like the proteinase K results, the treatment of the preformed biofilms with DNase I produced a very pronounced and significant (*p* < 0.001) decrease in the biofilm accumulated by HC474, the *lrgB* and *yycI* knockouts, and the trans-complementing mutants (Fig 4).

These results were also confirmed by CLSM experiments through which the effect of the DNase I treatment on the biofilm dispersal can be clearly visualized (Fig 4), indicating that, in addition to proteins, eDNA is a very important component of the biofilm matrix in the HC474 background. Accordingly, cell lysis-mediated eDNA release was subject to further investigation.

### Impact of the inactivation of *lrgB* and *yycI* in the autolysis of *S*. *aureus*

**Fig 5:**
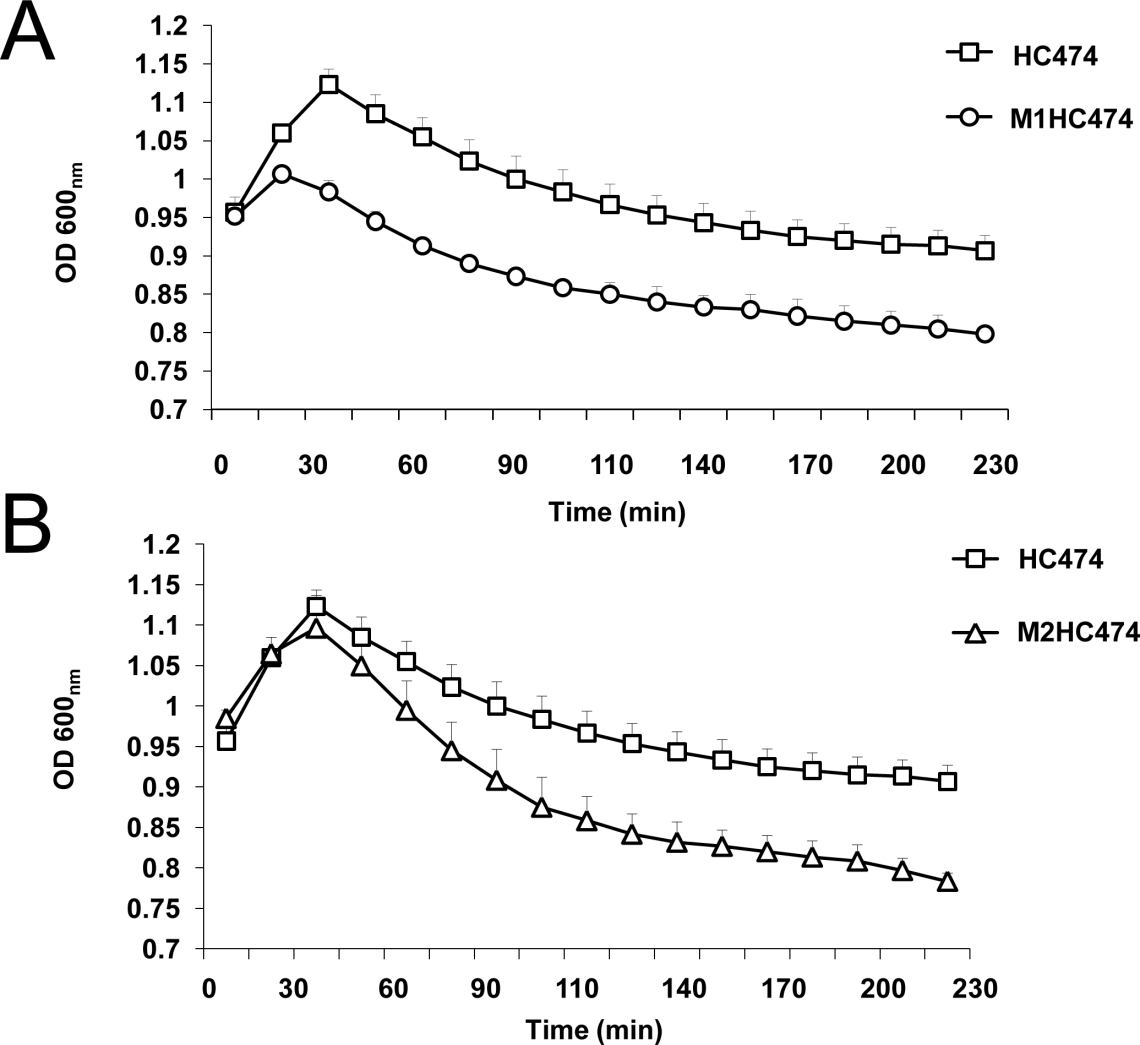
Autolytic activity of wild type (WT) strain HC474 and the isogenic (A) *lrgB* and (B) *yycI* knockouts in the presence of 0.1% Triton X-100. Cell lysis was measure at OD_600nm_ every 15 min until 4 h. The increase in the autolytic activity detected for both *lrgB* and *yycI* mutants were extremely significant ((p<0.0001).

The effect of *lrgB* and *yycI* disruptions in the autolysis of the HC474 strain was analyzed. Our data show that the inactivation of either *lrgB* (p<0.0001) or *yycI* (p<0.0001) lead to an extremely significant increase in the bacterial cell lysis (Fig 5A and 5B), when compared with the WT strain HC474, corroborating the hypothesis that increased cell lysis and eDNA content were involved in the enhanced biofilm accumulation detected for *lrgB* and *yycI* mutants.

### Role of eDNA in the increase of the biofilm formed by *lrgB* or *yycI* mutants

**Fig 6:**
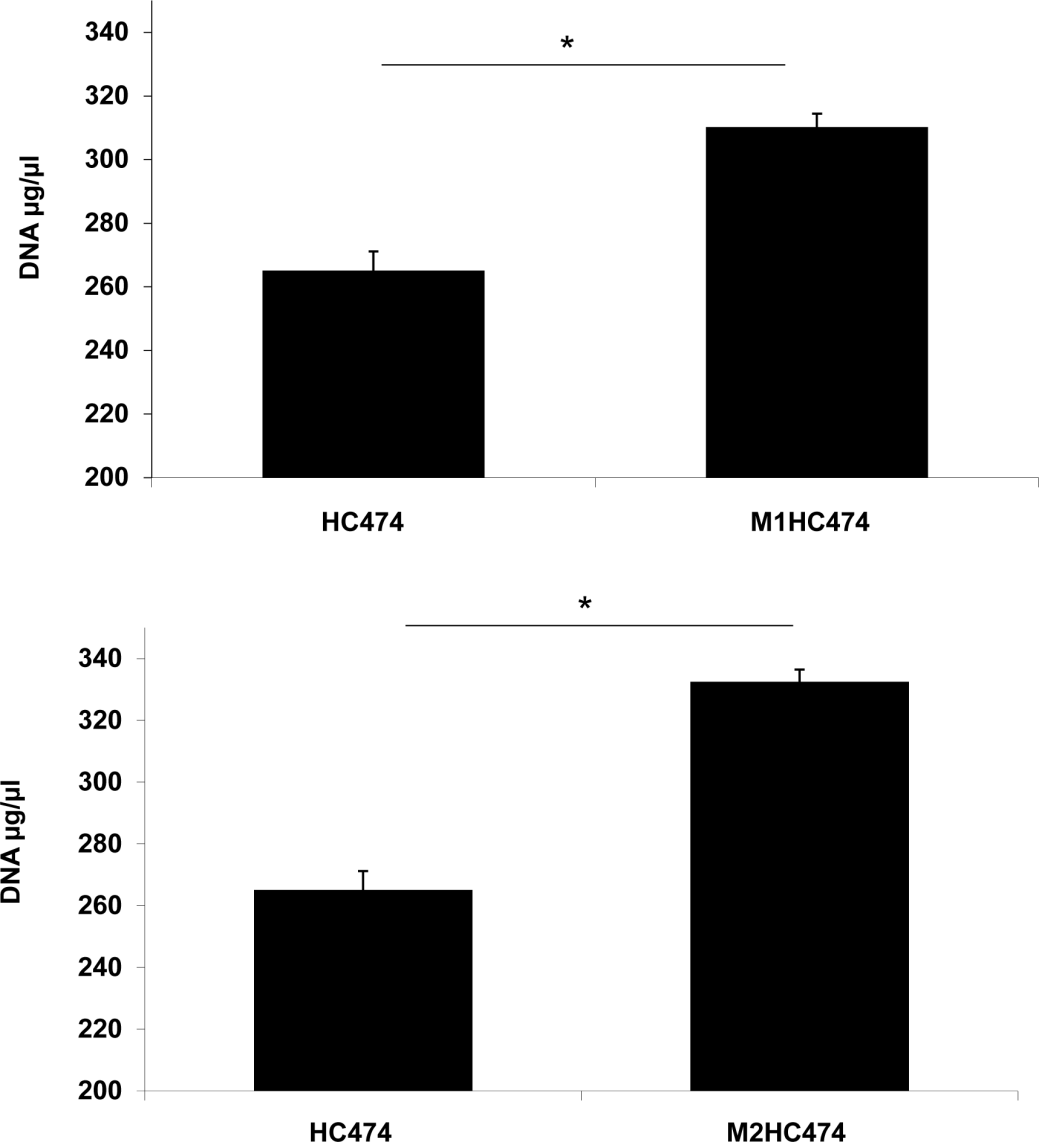
eDNA assay using wild type (WT) strain HC474 and the isogenic (A) *lrgB* and (B) *yycI* knockouts. The concentration of eDNA was determined in the biofilm supernatants (*p < 0.01).

To further confirm the involvement of cell-lysis dependent eDNA release in the knockout mutants, the content of eDNA was measured in the supernatants of the biofilms formed by *lrgB* and *yycI* knockouts and isogenic WT HC474. Accordingly, increased amount of eDNA was recovered from *lrgB* (*p* < 0.01) and *yycI* (*p* < 0.01) mutants compared with the WT strain (Fig 6A and 6B). Higher amount of eDNA was detected in biofilm supernatants of *yycI* knockout, which accumulates more biofilm than the *lrgB* mutant, suggesting a positive correlation between enhanced eDNA release and superior capacity for biofilm development.

### Transcriptional levels of the biofilm-associated *fnbAB* genes and the *rnaIII* and *sarA* regulatory genes in the *lrgB* or *yycI* knockouts

To examine whether the inactivation of *lrgB* or *yycI* would affect the expression of loci frequently associated with *ica*-independent biofilms (*fnbA*, *fnbB*, *sarA*, and *agr*-RNAIII), real-time qRT-PCR was performed using total RNA obtained from the in vitro sessile cells of the HC474, M1HC474, and M2HC474 strains. No significant differences were detected in the transcript levels for M1HC474 (RQ_*fnbA*_ = 1.34 ± 0.65; RQ_*fnbB*_ = 1.15 ± 0.44; RQ_*sarA*_ = 1.5 ± 0.45; RQ_agr-RNAIII_ = 1.1 ± 0.14) or M2HC474 (RQ_*fnbA*_ = 1.05 ± 0.35; RQ_*fnbB*_ = 1.32 ± 0.39; RQ_*sarA*_ = 1.70 ± 0.63; RQ_agr-RNAIII_ = 0.83 ± 0.14) relative to the HC474 parental strain (RQ = 1 for calibration; Figure A in S1 File).

### Expression of *lrgB* and *yycI* in biofilm environments

**Fig 7:**
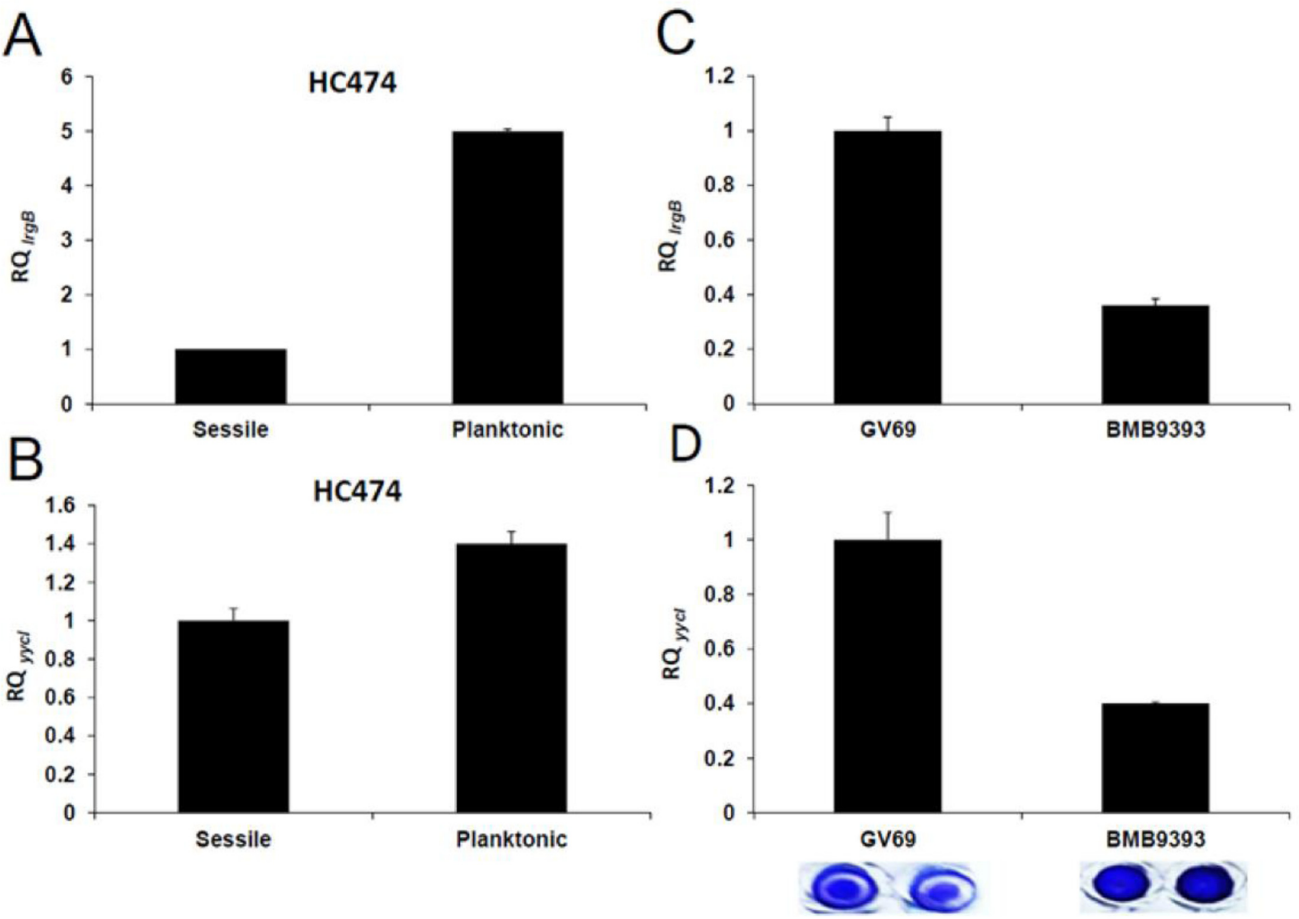
Transcriptional levels of *lrgB* and *yycI*. (A) and (B) Total RNA was isolated from free-living or sessile cells of the HC474 strain. (C) and (D) Total RNA was obtained from sessile cells of BMB9393 or GV69 isolates. Tests were performed using real-time qRT-PCR. The results were considered significant when the difference in gene expression varied by two-fold or more. RQ, Relative Quantity. Right bottom panel: Biofilm accumulated by the isolates BMB9393 (strong biofilm producer) and GV69 (moderate producer) on inert polystyrene microtiter plates.

Biofilm can represent a microniche where autolytic activity can be particularly regulated [34–36], thus we compared *lrgB* and *yycI* expressions of free-leaving cells to that of sessile cells of the HC474 strain. A strong attenuation in the expression of *lrgB* (5-fold decrease) was detected for sessile in relation to planktonic cells (Fig 7A). However, a small (lower than 2-fold) difference was detected in the transcriptional level of *yycI* when these two growth conditions were compared (Fig 7B).

Additionally, we tested a pair of MRSA clinical isolates from the same genotype that display biofilm phenotypes varying from very strong (BMB9393; biofilm value = 3.0) to moderate (GV69; biofilm value = 0.70; right bottom panel). In spite of the fact that these isolates are not isogenic, they are classified within the same MLST type (ST239) and *mec* type (SCC*mec* III), display the same pulsed-field electrophoresis band-pattern and are epidemiologically related [13]. The transcriptional level of both genes (*lrgB* and *yycI*) was more than 2-fold reduced for the strong biofilm producer BMB9393 when compared with GV69 isolate (Fig 7C and 7D). Despite the fact that the biofilm accumulated by both isolates could be importantly dispersed by DNase I, this enzyme removed 55.3% more biofilm produced by BMB9393 when compared to that developed by GV69 isolate (Data not shown).

## Discussion

Insertional inactivation by mobile genetic elements is a technique that has been used by several research groups to identify genes involved in *ica*-independent biofilm formation and/or accumulation [36, 37]. A classic example was the discovery of the role of the *bap* gene in the development of biofilm by an *S*. *aureus* isolate from bovine mastitis [37]. In our work, two genes were identified after mapping Tn*551* insertion sites in the chromosomes of HC474 mutants that showed increased biofilm accumulation. The *yycI* gene, an element of the TCS YycG/YycF (WalK/WalR) operon, also contains the *yycH* and *yycJ* genes, and *lrgB*, a component of the *lrgAB* operon, encodes proteins that seem to prevent homo-oligomerization of CidAB [34, 38, 39]. It is impressive that in both mutants, the Tn*551* disrupted loci that have recently been associated with autolytic systems. This was probably a consequence of the importance of eDNA in the development of HC474 biofilm, given that the DNase I treatment of the preformed biofilms by this strain and the derived knockouts almost completely disrupted them.

It has been proposed that *cidA* and *lrgA* encode peptides that show characteristics common to phage-holin and anti-holin, respectively, such as their small size, two or more transmembrane domains, and oligomerization properties [18, 20, 39]. Holin and anti-holin proteins are well characterized in certain bacteriophages and are known for their properties in regulating the timing of the phage lytic cycle [40, 41]. Studies have indicated that phage holin accumulates in the bacterial membrane to form a homo-oligomeric complex, leading to the formation of pores of different sizes depending on the holin, which allow autolysins to gain access to the bacterial cell wall, resulting in cell lysis. Alternatively, they cause a depolarization of the membrane potential with a consequent change of pH and the activation of pre-existing cell-wall peptidoglycan autolysins [41–43]. Accordingly, the anti-holin negatively regulates the holin-induced autolysis by specifically binding to the holin, thereby inhibiting its function. Generally, specific holin and anti-holin pairs are encoded by the same operon [41].

In this study, *lrgB* inactivation significantly increased the amount of biofilm formed by both the M1069 (Tn*551*::*lrgB*) and M1HC474 (Ω*lrgB*::pLGEM) mutants in relation to the WT HC474 strain. Furthermore, the superexpression of *lrgB* led to an inhibition of biofilm accumulation in comparison to WT. Mann and co-workers [21] demonstrated that the *lrgAB* double knockout, derived from the UAMS-I strain, also showed an increased ability to accumulate biofilm in vitro. In another study, Sharma-Kuinkel et al. [44] investigated the effect of the *lrgAB* positive regulator *lytSR* operon on biofilm development and reported that the UAMS-1Δ*lytS* mutant showed a stronger biofilm in vitro, probably by increasing cell lysis and the amount of eDNA in the biofilm matrix. Similarly, in our study, *lrgB* single inactivation also leads to an increased cell lysis and accumulation of eDNA in the biofilm. Even though *cidB* and *lrgB* are co-expressed with *cidA* and *lrgA*, respectively, their real functions in *S*. *aureus* are not completely understood. The few studies published thus far involved *cidAB* or *lrgAB* double knockouts or knockouts of the individual *lrgA* or *cidA* loci [18–21]. Despite this, it was proposed that the proteins codified by the *cidB* and *lrgB* loci might interact with CidA and LrgA, respectively, to exert their function as holins (CidA-CidB) or anti-holins (LrgA-LrgB) [34]. Like the *lrgAB* double mutant previously reported, our data showed that knocking out *lrgB* individually in the *S*. *aureus* clinical strain HC474 also promoted an increase in biofilm accumulation, indicating that, similarly to *lrgA*, *lrgB* is an important gene in this operon.

The other locus inactivated by the Tn*551* insertion was *yycI*. Previous studies of global gene expression have suggested that the YycG/YycF (WalK/WalR) TCS promotes the activation of nine genes involved in cell wall degradation [35]. In *Bacillus subtilis*, inactivating *yycH* and *yycI* resulted in the overexpression of genes upregulated by *yycG/yycF* (*walk/walR*), suggesting these proteins are negatively regulated by *yycHI* [45]. These proteins have an N-terminal transmembrane domain that may form a ternary complex with YycG (WalK), thereby preventing YycF (WalR) activation [45].

In this work, the *yycI*::Tn*551* and Ω*yycI*::pYGEM mutations in HC474 led to a significant increase in biofilm accumulation, while the overexpression of *yycI* reduced the biofilm development. In agreement with the findings for *B*. *subtilis*, it is possible that the absence of the YycGIH ternary complex in the insertional mutants leads to an upregulation of the murein hydrolases by the YycGF TCS and consequently to higher amounts of eDNA, resulting in the increased accumulation of biofilm. In fact, our data corroborate these assumptions, since *yycI* mutant showed increased autolytic activity concomitantly with the increase of the eDNA content. This is the first study to associate *yycI* with modulating biofilm formation in *S*. *aureus*. In a study with *S*. *mutans*, it was demonstrated that *yycJ* (*vicX*) inactivation, which also negatively regulates the YycGF TCS, led to an increase in biofilm accumulation [46]. Consistent with these data, Dubrac and co-workers [35, 38] demonstrated that increases in *yycF* expression resulted in increased biofilm accumulation by the *S*. *aureus* strain ST1000.

The importance of eDNA in biofilm formation and/or accumulation has been reported by several study groups [47–49], which have shown that eDNA has many functions that might contribute to the assembly of the biofilm architecture. It was suggested that eDNA may be involved in either the early stages of biofilm formation by promoting bacterial adhesion to the surfaces or in the biofilm maturation phase by promoting intercellular adhesion [50]. In addition, because its poly-anionic nature, eDNA can promote biofilm development by interacting with bacterial proteins (e.g. *S*. *aureus* beta-toxin) to form a nucleoprotein matrix [50–52]. Thus, it is possible that protein-eDNA interactions explain, at least in part, the strong effect of proteinase K in biofilms formed by HC474 and *lrgB*/*yycI* mutants. Some authors have also suggested that eDNA could assist biofilm stability by protecting sessile cells from the physical stresses and activity of antibiotics and detergents [51]. In fact, it was recently demonstrated that eDNA has a central role in biofilm development of about half of the MRSA clinical isolates from distinct genetic origins tested [53].

Thus, our data showed that in addition to eDNA, proteins are important key components of the biofilm produced by the HC474 strain. Surface proteins such as FnBPA and FnBPB have been considered important factors associated with *S*. *aureus ica*-independent biofilms [8, 9]. Thus, to investigate whether the increase in biofilm accumulation observed for the *lrgB* and *yycI* mutants could be correlated with a possible increase in *fnbAB* expression, the transcription levels of these genes were assessed by real-time qRT-PCR. Additionally, the expression of two global virulence regulators, *agr*-*rnaIII* and *sarA*, were also evaluated. No significant differences were detected in the expression of these genes in the mutants in comparison with the WT HC474 strain, ruling out their involvement in the increase of biofilm accumulation observed for the *lrgB* and *yycI* isogenic knockouts.

Under biofilm growth the WT strain HC474 had a strong attenuation in the *lrgB* expression, which is in agreement with the repression of biofilm development by this gene. Despite the fact we could not detected a statistically significant attenuation of *yycI* expression for HC474, the levels of *lrgB*/*yycI* transcription in the isolates BMB9393 and GV69, under biofilm growth, are in agreement with their biofilm formation capacity. However, a direct correlation would require inactivating these genes in the ST239 background. Similarly to the results observed for HC474, cell lysis-mediated eDNA release is likely to play a role in the biofilms formed by ST239 isolates, since DNase I causes biofilm dispersion. Taken all together, our data reinforcing the hypothesis that modulating autolytic system is part of important key mechanisms that ultimately determine whether or not a MRSA infection will move toward antimicrobial refractory and chronicity as a consequence of biofilm growth.

Chu and collaborators have demonstrated that mutation in *rot* (a global regulator of *S*. *aureus* exoproteins) enhances biofilm formation in *S*. *aureus* [54]. The transcriptional levels of autolysin encoding genes (*lytM*, *lytN*) and *lgrA* were affected by Rot directly binding to the promoter region of these genes [54]. Thus, it was concluded that Rot affects autolysis and biofilm accumulation by either directly regulating these autolysins or indirectly by downregulating *lrgA*, reinforcing the role of LgrAB system in biofilm development [54]. In addition to Rot, other *S*. *aureus* regulators (e.g., ArlS, SarA, SarV, and MgrA), well-known by their role in bacterial virulence, are also involved in the control of autolytic system and biofilm development [55–57].

Some authors have suggested a mechanism that induces programmed cell death (PCD) in bacteria that involves CidAB/LrgAB. Recently, an LRGB-like gene, similar to the bacterial *lrgB*, was found in the *Arabidopsis thaliana* chloroplast that was involved in the control of plant apoptosis, including mitochondrial death, and the activation of the caspase cascade, strongly suggesting that holin activity may also induce apoptosis in plants [58, 59]. Bayles has recently pointed out that the fact that mitochondria arose from bacterial endosymbiosis is strong evidence of a convergent evolution for PCD in plants and bacteria [60]. In the biofilm environment, bacteria can live in a customized microniche in which the occurrence of the controlled events of cell death would be fitting, since it would promote the increased availability of eDNA. It has been suggested that bacterial stress situations such as those occurring in the biofilm environment might trigger damage in the bacterial DNA molecule, leading to the activation of both the SOS repair system and the regulatory cascade activating PCD in a subset of bacterial cells [34, 60], again demonstrating the importance of autolytic mechanisms for biofilm accumulation and bacterial fitness.

These data led us to test the relevance of *lrgB* and *yycI* inactivation in a mouse model of foreign body infection. Corroborating the importance of the modulation of these genes over the course of an infection, the *lrgB* and *yycI* isogenic mutants showed a stronger ability to cause foreign body infections in mice as reflected by the higher number of sessile cells recovered from the catheter segments infected with these strains in comparison to those infected with WT.

## Conclusions

For the first time it has been clearly demonstrated that *lrgB* and *yycI* are important biofilm modulators, corroborating other studies that have highlighted the importance of autolytic mechanisms in the modulation/regulation of *S*. *aureus* biofilm. Most importantly, we showed that the inactivation of *lrgB* or *yycI* results in increased virulence in a murine foreign-body infection model. Therefore, the molecules involved in the mechanisms controlling cell death and autolysis may serve as potential targets for the development of anti-biofilm drugs.

## Supporting Information

S1 File **Table A**. Primers used in this study. **Figure A**. The transcription levels of *fnbA* (A and B), *fnbB* (C and D), *sarA* (E and F) and *agr-rnaIII* (G and H) in sessile cells as determined by real time quantitative RT-PCR. **ARRIVE checklist**.
(PDF)
